# A systematic review of validated assessments methods for head and neck lymphedema

**DOI:** 10.1007/s00405-023-07841-0

**Published:** 2023-02-10

**Authors:** Coralie R. Arends, Josephine E. Lindhout, Lisette van der Molen, Erica A. Wilthagen, Michiel W. M. van den Brekel, Martijn M. Stuiver

**Affiliations:** 1 Department of Head and Neck Oncology and Surgery, Netherlands Cancer Institute, Plesmanlaan 121, 1066 CX Amsterdam, The Netherlands; 2 Faculty of Health, Medicine and Life Sciences, Maastricht University, Maastricht, The Netherlands; 3 Amsterdam Center for Language and Communication, University of Amsterdam, Amsterdam, The Netherlands; 4 Scientific Information Service, Netherlands Cancer Institute, Amsterdam, The Netherlands; 5 Department of Oral and Maxillofacial Surgery, Amsterdam University Medical Center, Amsterdam, Netherlands; 6 Center for Quality of Life and Division of Psychosocial Oncology and Epidemiology, Netherlands Cancer Institute, Amsterdam, the Netherlands; 7 Center of Expertise Urban Vitality, Faculty of Health, Amsterdam University of Applied Sciences, Amsterdam, the Netherlands

**Keywords:** Head and neck cancer, Lymphedema, Validity, Reliability, Measurement

## Abstract

**Purpose:**

This systematic review aimed to provide a comprehensive overview of the validity and reliability of existing measurement instruments for quantifying head and neck lymphedema.

**Methods:**

Four databases were searched on January 31st, 2022. The COnsensus-based Standards for selecting health Measurement INstruments (COSMIN) checklists were used for the risk of bias (ROB) assessment.

**Results:**

Out of 3362 unique records, eight studies examined the reliability and validity of five measurement instruments of which one patient reported outcome. The Patterson scale for internal lymphedema and the patient reported head and neck external lymphedema and fibrosis (LIDS-H&N) demonstrated validity and reliability. For external lymphedema, none of the instruments had good reliability for all measuring points.

**Conclusion:**

There is a lack of sufficiently reliable and valid measurement instruments for external head and neck lymphedema. The Patterson scale and the patient reported LIDS-H&N seem reliable for clinical practice and research.

## Introduction

The often extensive treatment for head and neck cancer can cause long-term side effects. Well-known and extensively reported side effects include dysphagia [1, 2], xerostomia [3–5], and trismus [6–8], but lymphedema [9] is relatively understudied.

Head and neck lymphedema is a chronic accumulation of fluid and proteins in external structures (soft tissue) as well as in internal anatomical sites (mucous membranes and underlying soft tissues of the upper aerodigestive tract) [10, 11]. Head and neck lymphedema most commonly occurs when the lymphatic system is obstructed or disrupted due to surgery or (chemo-) radiotherapy [12]. About three in four patients treated for head and neck cancer (HNC) experience lymphedema [13, 14]. The presence of lymphedema can significantly impact patients’ quality of life [15–17]. It may cause pain; feelings of heaviness, tightness, or numbness; reduced mobility; and increased infection risk. Internal lymphedema can also affect articulation, voicing and can cause airway obstruction, obstructive sleep apnea, and swallowing difficulties. In addition, prolonged lymphedema can induce structural changes such as adipogenesis, fibrosis, and chronic inflammation [10, 11, 18–21].

Reliable screening, treatment, and follow-up of HNC-related lymphedema is important to optimize lymphedema care in head and neck cancer survivors. Therefore, the presence of a valid and reliable measurement instrument is needed to establish severity of lymphedema and to measure effectiveness of treatment. A few methods have been proposed and evaluated in terms of validity and reliability, and some external head and neck lymphedema measurement instruments have found their way into clinical practice [22–24]. Examples of these measurement instruments include clinician-administered subjective rating scales based on palpation of the head and neck, tape measurements, or tissue dielectric constant (TDC) measurements [22, 23]. For examining internal lymphedema, instruments such as endoscopy and imaging modalities are used; for example, sagittal computed tomography (CT) measurement of epiglottis thickness [24].

While there is consensus on how to measure and evaluate lymphedema of the limbs, this is not the case for head and neck lymphedema, and to our knowledge evidence based recommendations are currently lacking. This causes practice variation and lack of standardization of outcome measures in clinical studies. The purpose of this systematic review is to (1) provide a comprehensive overview of the literature on clinician-administered and patient-reported measurement instruments for the assessment of external- and internal lymphedema in the head and neck area in patients after HNC; (2) to determine the validity and reliability of the different methods; and (3) to provide recommendations for clinical practice based on evidence.

## Materials and methods

This systematic review was reported in adherence to the Preferred Reporting Items for Systematic Reviews and Meta-Analyses (PRISMA) [25]. The protocol for this review was registered with PROSPERO on April 28th 2020, and can be accessed via CRD42020168675.

### Literature search

We performed a systematic literature search, with the support of a medical information specialist (E.A.W.), in four electronic databases: MEDLINE (PubMed), Embase (OVID), SCOPUS, and PEDro. The last search date was January 31th, 2022. The search strategy included combinations of free-text keywords, equivalent words in title/abstract, and standardized keywords (MeSH and Emtree). The search strings were translated according to the standards of each separate database. Search terms included (“head and neck cancer” and “lymphedema”) or (“head and neck cancer” and “edema” and “reliability/validity”) (the full search strategy is shown in supplement 1), with no limits for date, study design, or language. Duplicate articles were removed according to the method of Bramer et al. [26]. In addition, we screened the reference lists of included studies and other reviews for potentially eligible publications that had not been identified during the initial search.

### Study selection

Studies that assessed the properties of measurement instruments for the severity of lymphedema in the head and neck area, either scored by clinicians or patient-reported (PROMs), were eligible for inclusion. Studies on patients with lymphedema due to non-oncological etiologies in the head and neck area or including only healthy participants were excluded. Two reviewers (C.R.A. and J.E.L.) independently screened the retrieved records based on title and abstract, blinded to each other, using Rayyan QRCI [27]. Next, full-text screening, also done blinded by these two reviewers, was done using EndNote [Clarivate analytics, Philadelphia, United States]. Disagreements in either selection step were resolved through consensus meetings.

### Data extraction

To extract the data from each study, a structured data collection form was used. Data extraction included: sample size, study population, name of the measurement tool/technique assessed, reported results of intra- and interrater reliability, and validity indices.

### Risk of bias assessment

The COnsensus-based Standards for selecting health Measurement INstruments (COSMIN) risk of bias (ROB) checklist was used for the risk of bias assessment of the PROM studies [28–30]. For studies on clinician-rated instruments, we used the COSMIN Risk of Bias tool for assessing the quality of studies on reliability and measurement error of outcome measurement instruments [31]. The COSMIN tools are designed to support the selection of the most suitable outcome measurement instrument. The COSMIN consists of several “boxes” related to aspects of reliability and validity testing. As recommended by the authors of the COSMIN tool, only the boxes that were relevant to the study of interest were scored. Per box, a study could score +  + (very good); + (adequate); ± (doubtful); or − (inadequate). To determine the overall quality of a study, the lowest rating of any standard in the box was taken (i.e., “the worst score counts” principle), as suggested by COSMIN. Two independent and blinded researchers (C.R.A. and J.E.L.) and scored all included articles. Disagreements in scores were resolved by consensus, and if needed, by consulting a third reviewer (M.M.S).

## Results

### Results of the search

**Fig. 1:**
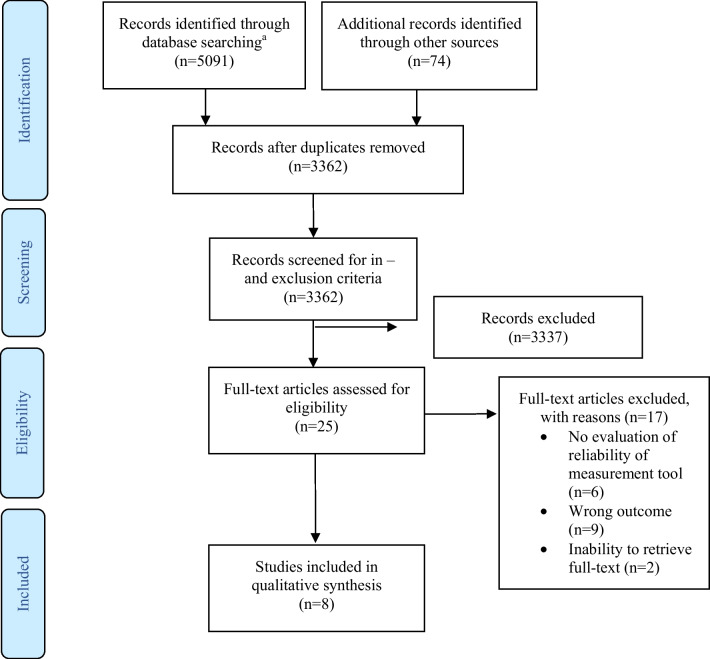
PRISMA flow diagram. ^a^MEDLINE (PubMed), Embase (OVID), SCOPUS, and PEDro

Our systematic search identified 5091 records, and an additional 74 records were identified through backward and forward reference checking. After resolving duplicates, 1803 records, the in- and exclusion criteria were applied to 3362 abstracts. In this step, 3337 articles were excluded and we retrieved the remaining 25 articles for full-text. Of these, eight articles explicitly reported the reliability and validity of measurement instruments for lymphedema in the head and neck area and were included in the final analysis. Figure 1 illustrates the process used for the search. Due to the limited number of available articles and variance between measuring instruments and reported outcome measures addressed in those studies, no meta-analysis was possible. We provide a narrative summary of the included studies.

### Study characteristics and quality of the evidence

The included articles were published between 2007 and 2021. Seven of the eight studies were single-center cross-sectional studies. Only the study of Deng et al. was a prospective study, with six follow-up moments [32]. The number of included participants varied between 7 and 117 (Table 1). Three studies included healthy subjects as a control group [33–35].

**Table 1:** Characteristics of included studies and instruments assessed

Study	*n*	Population	Measurement	Instrument type	Raters	Reliability assessment	Hypothesis testing
Chotipanich and Kongpit (2020) [33]	100	50% HNC patients 50% healthy controls	Tape measurement	7 key facial distances, 2 facial circumferences, and 3 neck circumferences	20	ICC interrater: facial distances 0.33–0.70 (*S*_w_ 4.6–8.8 mm), facial circumferences 0.70 and 0.81 (*S*_w_ 11.8 and 18.3 mm), neck circumferences 0.90–0.95 (*S*_w_ 8.3–12.3 mm)	–
Deng et al. (2015a, b) [37]	30	HNC patients > 3 months after treatment	HN-ELAF	Physical examination grading lymphedema (grade 1–5) per location	2	Interrater: 83% agreement (kappa 0.75, p < 0.001) and concordance correlation coefficient 0.91	–
Deng et al. (2016a, b) [36]	60	HNC patients > 3 months after treatment	HN-LEF	Physical examination grading lymphedema (type and grade)	2	Interrater: type agreement ranged from 78.8% (submental) to 98.1% (left supraclavicular fossa). Grade agreement ranged from 68.2% to 100% Intrarater: type agreement ranged from 92.6% (submental) to 97.2% (supraclavicular fossa). Grade agreement was 91.4% across all sites	Ultrasonography assessment supports all three hypotheses in the submental, cheek, and neck regions. Lack of correlation in the peri-orbital and supraclavicular region
Deng et al. (2020) [32]	117	HNC patients	LSIDS-H&N	64-item patient-reported outcome measure	–	Explored 7 symptom domain clusters with good internal consistency. Variating with a Cronbach’s alpha of 0.49 to 0.90	–
Patterson et al. (2007) [35]	25	23 HNC patients 2 healthy volunteers	Rating scale of endoscopic video images	11 structures and 2 spaces were scored as normal, mild, moderate, or severe	5	Interrater mean weighted kappa 0.54 (range 0.35–0.70) Intrarater mean weighted kappa 0.84 (range 0.72–0.91)	–
Purcell et al. (2016) [34]	40	50% HNC patients 50% matched healthy controls	ALOHA measurement protocol	4 tape measurement points, and MMD	3	ICC interrater: for MMD was 0.973, tape measurement variated between 0.420–0.979 ICC Intrarater: for MMD of 0.974	Significant for MMD and upper neck circumference with tape measurement. The correlation between MMD and MD Anderson levels was moderate (rho = 0.587)
Ridner et al. (2021) [39]	72	HNC patients	LSIDS-H&N	48-item patient-reported outcome measure	–	Explored 7 symptom clusters. Cronbach’s alpha varied from 0.83 to 0.95	Validity pattern with the FASQ, PROMS-SF, LyQLI, and the Marlowe–Crowne. Not all confirmed
Starmer et al. (2021) [38]	7	Video samples HNC patients	Revised Patterson Edema Scale	8 structures were scored as normal, mild, moderate, severe, or not evaluable	28	Overall interrater kappa across raters was 0.64. Reliability improved comparing the original scale except for aryepiglottic folds and true vocal folds	–

*n* sample size, *HNC* head and neck cancer, *HN-ELEF* Head and neck External lymphedema and fibrosis assessment criteria, *HN-LEF* Head and neck External lymphedema and fibrosis assessment criteria, *ALOHA* Assessment of Lymphedema of the Head and Neck, *LSIDS-H&N* Lymphedema Symptom Intensity and Distress Survey-Head and Neck, *ICC* Intraclass Correlation Coefficient, *S*_*w*_ within-subject standard deviation, *MMD* MoistureMeter D, *FASQ* Functional Assessment Screening Questionnaire, *PROMS-SF* Profile of Mood States-Short Form, *LyQLI* Lymphedema Quality of Life Inventory

Four studies used an instrument for assessing *external* lymphedema; all were clinician-rated [33, 34, 36, 37]. These instruments consisted of assessment with a tape [33, 34], the MoistureMeterD (MMD) [34], the Head and Neck External Lymphedema and Fibrosis (HN-ELAF) assessment Criteria [37], and the Head and Neck External Lymphedema and Fibrosis (HN-LEF) Assessment Criteria [36], respectively. Two studies concerned the assessment of *internal* lymphedema, using a rating scale on laryngopharyngeal video endoscopic images[35, 38]. In the two remaining studies, both done by the same research group, the development and evaluation of a PROM on head and neck lymphedema, the Lymphedema Symptom Intensity and Distress Survey-Head and Neck (LSIDS-H&N) [32, 39] was evaluated. Measurement properties examined included interrater reliability (*n* = 6) [33–38] and intrarater reliability (*n* = 3) [34–36].

For ROB assessment we used the COSMIN tools. Content validity of the LSIDS-H&N was assessed in one study, by Deng et al. This study received a score of ‘doubtful’, because it was unclear whether there were at least two researchers involved in the analysis [32]. Two studies assessed structural validity and internal consistency of LSIDS-H&N. Both were scored ‘inadequate’ for the assessment of structural validity, because of the small sample size, but ‘very good’ for the assessment of internal consistency [32, 39]. Three studies assessed construct validity using hypotheses testing, for the LSIDS-H&N, MoistureMeterD, tape measurement, and the HN-LEF, respectively [34, 36, 39]. Risk of bias was scored as ‘doubtful’ for all these studies because it was unclear whether the hypotheses tested were formulated a priori, because the hypotheses were first mentioned in the results or discussion section and not in the method. Also, the sample size calculations were not based on hypothesis testing, while conclusions on construct validity were based on statistical significance. For assessment of reliability [33–38] and measurement error [33, 36–38], the scores varied between ‘doubtful’ and ‘very good’. Full details of the risk of bias assessment of each study is reported in Table 2.

**Table 2:**
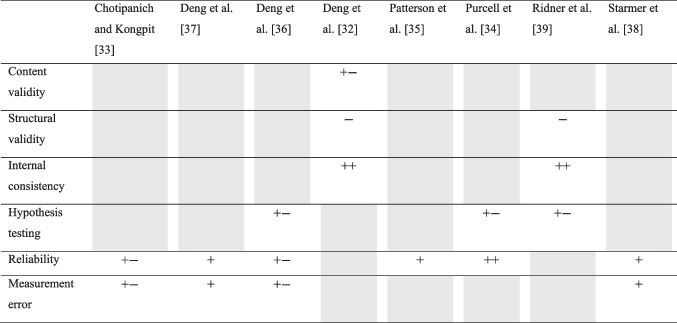
COSMIN checklist for ROB assessment

ROB, risk of bias; ++, very good; +, adequate; +−, doubtful; −, inadequate; grey: not assessed in the study under scrutiny

### Reliability and validity of the measurement instruments

#### External lymphedema

##### MoistureMeterD

The measurement properties of the MoistureMeterD, a device estimating extracellular water percentage using dielectric constant analysis, was studied by Purcell et al. using a single submental measuring point. The interrater reliability was excellent (ICC 0.973) [34]. They also compared the head and neck lymphedema group to a matched healthy control group, which showed a significant difference between the groups for the MoistureMeterD with a standardized mean difference of 2.84. Furthermore, the authors reported a significant correlation (rho = 0.587) between MoistureMeterD and the MD Anderson head and neck lymphedema rating scale, as evidence supporting validity.

##### Tape measurements

Two studies used tape measurements to quantify lymphedema [33, 34]. Chotipanich and Kongpit used tape measuring for seven key facial distances, two facial circumferences, and three neck circumferences. The reliability of the facial distance measurements varied from poor (ICC 0.33) to good (ICC 0.70) The two facial circumferences showed good reliability with ICCs of 0.70 (vertical, in front of the ears) and 0.81 (diagonal, chin to crown of the head), respectively. The reliability of neck circumference measurement points was good (ICC 0.90, inferior neck) to excellent (ICC 0.95; middle neck) [33]. Purcell et al. reported high reliability for measurements of ear to ear distance (ICC 0.948), upper neck circumference (ICC 0.969), lower neck circumference (ICC 0.979), but low reliability for lip to lower neck circumference distance (ICC 0.420). There was no correlation (variating between − 0.16 and 0.263) between the tape measurements and the MD Anderson scale, suggesting inadequate validity [34].

##### Head and neck lymphedema and fibrosis assessment criteria

The HN-ELAF criteria classify lymphedema and fibrosis into four phenotypes, dependent on palpable thickening, tightness of the dermis, visible swelling, reducibility and persistency. The HN-ELAF showed an interrater reliability absolute agreement of 83%, with a kappa 0.75, *p* < 0.001, in one study [37]. The HN-ELAF criteria were subsequently revised and renamed HN-LEF. The HN-LEF criteria showed interrater reliability with a kappa ranging from 0.69 (submental) to 0.95 (left supraclavicular fossa). Intrarater reliability varied with a kappa of 0.18 (left peri-orbital) to 1.00 (cheek) [36].

For validity assessment, Deng et al. grouped ultrasound measurements in five regions (peri-orbital, cheek, submental, neck, and supraclavicular fossa) corresponding with the HN-LEF sites. The area under the curve (AUC) from the ultrasound value on each region was used as the reference standard to enable known group comparisons. Known group validity was largely confirmed for the submental, cheek, and neck regions, but expected associations of the peri-orbital and supraclavicular regions with HN-LEF typing was not confirmed [36].

#### Internal lymphedema

The two studies on internal lymphedema assessed the measurement properties of the Patterson scale and the Revised Patterson Scale, respectively [35, 38]. Both instruments use images obtained via flexible laryngoscopy.

The revised Patterson Scale has been shortened, and to improve reliability, a description for each severity level was added with photographic examples. These additions improved the weighted kappa for the epiglottis (0.63 to 0.78), vallecula (0.34 to 0.68), pharyngoepiglottic folds (0.40 to 0.52), arytenoid (0.65 to 0.69), false vocal fold (0.61 to 0.66) and pyriform sinuses (0.53 to 0.54). The aryepiglottic folds (0.65 to 0.63) and the true vocal folds (0.43 to 0.23) showed less favorable agreement compared to the first study. The mean weighted kappa was 0.54 in the original scale, and 0.64 for the revised scale [35, 38].

#### Patient reported outcome measures (PROMs)

A single PROM was identified, reported in two studies. Deng et al. developed the LSIDS-H&N, a patient reported outcome for head and neck lymphedema and fibrosis. They started with 64 items, which were eventually reduced to 33 items in seven symptom domain clusters with good internal consistency; Cronbach’s alpha for each cluster ranged between 0.49 and 0.90 [32].

In a subsequent study, Ridner et al. revised the LSIDS-H&N to include 48 items in seven clusters. The Cronbach’s alpha for the clusters varied between 0.83 and 0.95. Construct validity of the scale was assessed by correlating the LSIDS-H&N cluster scores to other questionnaires [39]. Out of a total of 10 hypothesis, 7 were mostly confirmed. This included, for example, a low correlation with the Marlowe-Crowne Social Desirability Scale for all clusters; a strong inverse correlation of the activity cluster with the Functional Assessment Screening Questionnaire (FASQ); and a strong correlation of the Profile of Mood States—Short Form (PROMS-SF) scores on vigor and fatigue with the *Activity* cluster. Also, scores on Lymphedema Quality of Life Inventory (LyQOLI) correlated with the practical cluster strongest with the clusters *Activity* and Sexuality, as hypothesized.

*n* sample size, *HNC* head and neck cancer, *HN-ELEF* Head and neck External lymphedema and fibrosis assessment criteria, *HN-LEF* Head and neck External lymphedema and fibrosis assessment criteria, *ALOHA* Assessment of Lymphedema of the Head and Neck, *LSIDS-H&N* Lymphedema Symptom Intensity and Distress Survey-Head and Neck, *ICC* Intraclass Correlation Coefficient, *S*_*w*_ within-subject standard deviation, *MMD* MoistureMeterD, *FASQ* Functional Assessment Screening Questionnaire, *PROMS-SF* Profile of Mood States-Short Form, *LyQLI* Lymphedema Quality of Life Inventory

## Discussion

The purpose of this systematic review was to provide a structured overview of the literature on the validity and reliability of measurement instruments for the assessment of external—and internal lymphedema in the head and neck area. In total, eight studies met the inclusion criteria. These studies were scored on their ROB and results were summarized. The measurement tools identified were tape measurements, the MoistureMeterD, the clinician rated HN-LEF criteria for external lymphedema, the Patterson scale for internal lymphedema, and a single PROM: the LSIDS-H&N.

### Quality and completeness of the evidence

In an earlier ‘state of the art’ scoping review on measurement instruments for head and neck lymphedema, by Deng et al., the authors pointed out that there was a lack of reliable measurement tools and that adequate measuring of lymphedema in the head and neck area remained a challenge [40]. Following this publication, several studies were published on this topic. A systematic review by Tyker et al. on the treatment of lymphedema after HNC included twenty-six articles, in which a wide variety of measurement instruments were used to assess treatment results. Unfortunately, these measurement instruments were insufficiently validated for the head and neck area, which hindered a comparison of differences in treatment effects. In the studies included in that review, the reliability of instruments in other body parts was often extrapolated to the head and neck area, which may not be warranted. The authors of this review again emphasized the need for a reliable measurement tool which could be implemented in clinical practice and used consistently in research [13].

Based on the findings of our review, this challenge is still unmet for measurement instruments assessing external lymphedema. While tape measurement, the MoistureMeterD, and the HN-LEF have all shown some promising results for some, but not all, measuring locations in the head and neck area, reliability of each of these instruments could be further improved, and evidence for validity is still limited at best. With regard to measuring internal lymphedema, the revised Patterson scale showed a moderate to good interrater reliability except for the true vocal folds, and thus seems adequate for clinical practice and research.

The only patient-reported outcome identified; the LSIDS-H&N, showed good reliability in two studies, and some evidence to support validity.

Quality of study design and reporting could be improved in future studies, as evidenced by the high number of ‘doubtful’ scores assigned to the studies included in this review. For example, three studies did not describe their hypothesis in the methods, but rather in the results or discussion section. As a result, it is unclear if these were a-priori or post-hoc hypotheses [34, 36, 39].

### Implications for practice and research

Standardization of measures for assessment of external lymphedema in the head and neck area in clinical practice would be highly desirable. Nevertheless, the current evidence is still too limited to recommend any single instrument for this purpose. Given the small number of studies and the methodological limitations in many of them, further studies are needed to strengthen the evidence base for existing measurement instruments as well as to improve the reliability of measurement procedures. Future studies would benefit from careful design and reporting, taking into account all quality recommendations for conduct and reporting of clinimetric research as proposed by COSMIN [41].

The revised Patterson scale seems adequate for assessing internal lymphedema in clinical practice and research. To this end we would recommend to make recordings when laryngoscopies are conducted in daily practice, making sure that the anatomical locations used in the Patterson scale are clearly visualized, and to save the images in the electronic hospital records for future reference and research.

Although the validity of the LSIDS-H&N requires further confirmation, this patient reported outcome measure is the only one currently available. Since it has sufficiently promising measurement properties, it could be considered for use in clinical practice when appropriate.

### Strengths and limitations of this review

This systematic review was done according to rigorous methodological standards: two independent authors conducted all steps of the systematic review (C.R.A. and J.E.L.), and no constraints were put on language, resulting in a broad search with a limited risk of missing relevant publications. Unfortunately, for two articles, no full text could be obtained, which is a limitation of our results. The quality rating was based on reported information. Due to shortcomings in meeting reporting standards, our ROB assessment may have been overly strict, however, we preferred to err on the side of caution instead of relying on benefit of the doubt. Finally, a meta-analysis of the reliability of the measurement instruments was not possible due to the heterogeneous nature of the studies and limited available data.

## Conclusion

To date, no single clinician-rated measurement instrument for external lymphedema can be recommended for clinical practice without caution due to limitations in reliability and heterogeneity in the applied measurement protocols. The MoistureMeterD is reliable for the measurement of extracellular water in the submental region. The only currently available PROM—the LSIDS-H&N—has shown promising reliability and can be used in clinical environments. The revised Patterson scale is very promising for measuring internal lymphedema and seems to be useful in clinical practice.

## Data Availability

Data are available upon reasonable request from the corresponding author.
